# Characterization of vaccine confidence among teachers in British Columbia, Canada: A population-based survey

**DOI:** 10.1371/journal.pone.0288107

**Published:** 2023-07-12

**Authors:** C. Sarai Racey, Robine Donken, Ellie Fox, Imogen Porter, Julie A. Bettinger, Jennifer Mark, Lizl Bonifacio, Meena Dawar, Mike Gagel, Rakel Kling, Silvina Mema, Hana Mitchell, Ian Roe, Gina Ogilvie, Manish Sadarangani

**Affiliations:** 1 School of Population and Public Health, Faculty of Medicine, University of British Columbia, Vancouver, BC, Canada; 2 Women’s Health Research Institute, British Columbia Women’s Hospital and Health Centre, Vancouver, BC, Canada; 3 Vaccine Evaluation Center, BC Children’s Hospital Research Institute, Vancouver, BC, Canada; 4 Department of Pediatrics, Faculty of Medicine, University of British Columbia, Vancouver, BC, Canada; 5 Vancouver Costal Health Authority, Vancouver, BC, Canada; 6 Northern Health Authority, Prince George, BC, Canada; 7 Interior Health Authority, Kelowna, BC, Canada; 8 BC Children’s Hospital Research Institute, Vancouver, BC, Canada; 9 BC Centre for Disease Control, Vancouver, BC, Canada; University of Ilorin, NIGERIA

## Abstract

**Objectives:**

Teachers are an important occupational group to consider when addressing vaccine confidence and uptake for school-aged children due to their proximate role within school-based immunization programs. The objectives of this study were to characterize and identify sociodemographic factors associated with vaccine confidence and describe teachers’ knowledge of and perceived role in the school-based immunization program, with the aim of informing public health policy and identifying opportunities for supporting teachers in their role in school-based immunization programs.

**Methods:**

A cross-sectional survey of elementary and secondary public-school teachers in British Columbia was completed from August to November 2020. Respondents provided sociodemographic information, as well as past vaccination experience, vaccine knowledge, and perceived role in the school-based immunization program. Vaccine confidence was measured using the Vaccine Hesitancy Scale (VHS). Characteristics associated with the VHS sub-scales ‘lack of confidence in vaccines’ and ‘perceived risk of vaccines’, were explored using ANOVA. Descriptive analysis was completed for teachers’ perceived role in the immunization program.

**Results:**

5,095 surveys were included in this analysis. Overall vaccine confidence was high, with vaccine hesitancy being related to the perceived risk of vaccines rather than a lack of confidence in the effectiveness of vaccines. ANOVA found significant differences for both VHS-sub-scales based on sociodemographic factors, however, the strength of the association was generally small. High general vaccine knowledge and never having delayed or refused a vaccine in the past were associated with higher vaccine confidence. Overall, teachers reported a lack of clarity in their role within the school-based immunization program.

**Conclusions:**

This large population-based observational study of teachers highlights a number of key engagement opportunities between public health and the education sector. Using a validated scale, we found that overall, teachers are highly accepting of vaccines, and well situated as potential partners with public health to address vaccine hesitancy.

## Introduction

Vaccine hesitancy has been defined as ‘*a delay in acceptance or refusal of vaccines despite availability of vaccine services*. *Vaccine hesitancy is complex and context specific*, *varying across time*, *place and vaccines’* [1]. In 2019, the World Health Organization (WHO) declared vaccine hesitancy as one of the top ten threats to global health, alongside climate change and antimicrobial resistance [2].

In Canada, publicly funded vaccines are provided by provincial public health immunization programs, with recommended vaccine schedules varying by province. In British Columbia (BC), publicly funded vaccines are provided through local public health or primary care clinics for pediatric vaccines administered up to 4 to 6 years of age [3]. The public health delivered school-based immunization program administers the majority of vaccines to school-age children in grade 6 (11–12 years old) and grade 9 (14–15 years old) (see supporting information). The grade 6 program (11-12-year-olds) offers HPV vaccine (2 doses) and catch-up programs for hepatitis B and varicella vaccines, with the grade 9 program offering meningococcal (quadrivalent) vaccine and Tdap (tetanus, diphtheria and pertussis) vaccine and catch-up program for HPV. The school-based immunization program is how the majority of vaccines are delivered to children >7 years of age. The exceptions are the annual influenza vaccine, and COVID-19 vaccines, which are delivered through local public health units, pharmacies, primary care clinics or mass immunization clinics.

Immunization delivery within school optimizes access by leveraging the presence of eligible youth in same space and time. This enables efficient program delivery by public health and reduces the burden on parents or guardians to facilitate youth attending immunization clinics. In addition, public health has the capacity to employ mature minor consent, which is authorized under the Infant Act legislation, and is a process by which a child under the age of 19 may consent to receive health care if they have been assessed by a healthcare provider to be competent to provide consent for themselves, independent of their parents’ or guardians’ wishes [4]. At present, public health typically only employs mature minor consent for immunizations with adolescent youth.

Despite the low barrier nature and public funding of the school-based immunization program, there continues to be an urgent need to identify opportunities and facilitators to increase vaccine uptake in school-aged youth. Provincially, acceptance of human papillomavirus (HPV) vaccine is particularly low, with 67% of grade 9 students having been immunized against HPV in 2019, whereas other vaccines have coverage rates that vary between 80% - 90% or greater [5].

Teachers have an influential position within the social fabrics of families and their students. Studies of school-based HPV vaccination programmes indicate that teachers’ knowledge, attitudes and beliefs towards vaccination can be an important factor influencing vaccine acceptance and uptake in students [6].

There is currently limited information on the general characterization of vaccine confidence in public-school teachers, with preliminary findings indicating the overall teachers are supportive of vaccination [7, 8], As such, there continues to be a need to measure and characterize vaccine confidence, explore how teachers perceive their role within the school-based immunization program, and identify potential for opportunities to engage teachers more in school-based immunization programs. Increasing our understanding of vaccine confidence, in relation to the role teachers play in immunization decision making, will inform potential future interventions to improve vaccine uptake.

The objectives of this study were to characterize vaccine confidence among public school teachers, identify sociodemographic factors associated with vaccine confidence, and to describe teachers’ knowledge of the school-based immunization program and their perceived role within the program. The aim of this exploratory study is to inform public health programs by identifying opportunities to engage and support teachers within the school-based immunization program.

## Materials and methods

### Study design

A cross-sectional survey of elementary and secondary public-school teachers in BC was completed from August 20^th^—November 3^rd^, 2020. This study was approved by the University of British Columbia Children’s and Women’s Research Ethics Board (#H20-01820)

### Participant recruitment

All teachers who had an email address on public school district website (between June—July 2020) were invited to participate in an online survey using individual email invitations through the secure REDCap survey platform [9]. Administrative and non-teacher support staff were excluded, based on school website information. Respondents were provided study information, completed an electronic informed consent, and were invited to participate in a draw for gift cards (one of 20, $100 gift cards). A maximum of two reminder invitations were sent one week apart to non-responders.

### Response rate

Response rate (%) was the sum of completed surveys (as indicated by the respondent submitting the survey as complete) plus partial surveys of those who clicked on the survey link but did not submit the survey, divided by the number of invitations sent to respondents with valid email addresses, as per the American Association for Public Opinion Research guidelines [10]. Email addresses that were auto-returned as undeliverable, or no longer active, were considered invalid. A minimum sample size of 2,400 completed surveys was needed to provide 95% CI within a 2% margin of error for population proportion estimates of 0.5 in our descriptive analysis. It was estimated that the response rate to an anonymous online survey would be limited, and so all identified eligible teachers were sent an invitation. Representativeness of respondents was compared to the distribution of teachers in BC based on age, sex, and population distribution of the five provincial health authorities [11–13].

### Survey instrument design

The survey was developed using existing literature and previous surveys, in addition to input from experts on vaccine confidence and acceptability (MS, GO, JAB, HM) (see Supporting information). Sociodemographic items included age, sex, gender, educational training, and length of time of residence in BC, as well as geographical location based on self-reported current school district of employment at time of the survey. Indigenous ancestry and visible minority categories were based on the Statistics Canada 2016 census [14].

Respondents were asked about past vaccination experience, and sources for general vaccine information (e.g., public health, government websites, school district or BC teachers’ union, general news sources, social media, friends or colleagues). General vaccine knowledge was measured using a modified scale [15], and respondents were also asked to identify which vaccine preventable diseases are included in the school-based immunization schedules for kindergarten school entry, grade 6, and grade 9 in BC. Participants were specifically asked about their awareness and knowledge of mature minor consent [4].

Vaccine confidence was measured using the validated 9-item Vaccine Hesitancy Scale (VHS), which was developed by the WHO’s Strategic Advisory Group of Experts (SAGE) Working Group on vaccine hesitancy [16, 17], and has been validated in a number of different populations and settings [18–20].The VHS items are measured using a 5-point Likert scale with responses from strongly disagree to strongly agree. Low scores on the VHS corresponds with low vaccine hesitancy and correspondingly high vaccine confidence [16, 17].

### Statistical analysis

General vaccine confidence was characterized using the VHS based on the two-sub-scales presented by Shapiro et al. 2018 [16]. Mean scores for both sub-scales of the VHS were calculated and analyzed separately, which included a 7-item ‘*lack of confidence in vaccines’* sub-scale (reverse scored), which measured beliefs about vaccine effectiveness and if vaccines are important for health (e.g., Childhood vaccines are effective) and the 2-item ‘*perceived risk of vaccines’* sub-scale, which measured beliefs about the risk and safety of vaccines [14] (e.g. new vaccines carry more risk than older vaccines), with lower scores indicating higher vaccine confidence. Cronbach’s alpha of >0.6 was used to assess reliability of both sub-scales.

The VHS is a validated measure of vaccine hesitancy, however, as an acknowledgement that vaccines are in fact accepted amongst the majority of populations and to use more affirmative terminology, we will therefore be using the VHS to measure general vaccine confidence. The measure of general vaccine confidence in this analysis is distinct from the VHS’s subscale “lack of confidence in vaccines”, and reflects the overall measure of general vaccine confidence as measured by the entirety of the VHS.

General vaccine knowledge was measured using an adapted one-dimensional vaccine knowledge scale [15], based on a grand score on 5-items using KR-20 for reliability (>0.6). The general vaccine knowledge was then categorized based on the score: high (4.0 or >), medium 2.1–3.9, and low (2.0 or <).

Reliable sources of information on vaccination were defined as public health, government websites, professional organizations (teachers’ union and school boards), and/or health care providers.

Teachers’ knowledge of the school-based immunization program and their perceived role in the school-based immunization program was summarized descriptively and categorized based on if they reported having taught grade 6 or grade 9 in the past 5 years, given that the BC school-based immunization program is delivered in those grades. Fisher’s exact tests (p<0.05) were used to evaluate differences in perceived role based on teaching history.

Characteristics associated with both VHS sub-scales, ‘*lack of confidence in vaccines’* and ‘*perceived risk of vaccines’*, were explored using ANOVA, with an effective size calculated to quantify the strength of the association (eta squared, η^**2**^) for those characteristics that were significant (p<0.05). Case-wise deletion was used for missing data.

Differences in mean scores of the VHS based on if respondents reported they believed it was appropriate for them to share beliefs about vaccines with students and parents was also explored, with those who responded agree or strongly agree to the 5-point Likert scale classified as endorsing the sharing of personal opinions as a teacher. All analyses were completed in R 4.0.1 [21].

## Results

A total of 29,184 email addresses were identified on public-school board websites for the estimated 45,000 BC public school teachers [12]. All were sent email invitations, of which 1,072 were deemed invalid due to automated undeliverable or inactive accounts. Of the 28,112 teachers with valid email addresses, we received 5,725 responses, with 5,095 (88.9%) having submitted a complete survey, which exceeded our minimum sample size required. Respondents were recorded from 58/60 public school boards across the province and were representative based on sex and age distributions to most current occupational data from the Ministry of Education [13], and aligned with the population distribution among the five geographical health regions for BC [22].

### Participant characteristics

Of the 5,095 respondents, 74.8% identified as female, and <1% identified as gender diverse (Table 1), with 16.2% identifying as a visible minority, and 23.5% reporting their formal education was in a scientific discipline (science or engineering).

**Table 1:** Summary of participant characteristics and association with vaccine hesitancy as measured by ANOVA of the two sub-scales of the Vaccine Hesitancy Scale (VHS).

**Participant Characteristics**	**N**	**%**	**VHS Factor 1:** *Lack of confidence in vaccines*				**VHS Factor 2:** *Perceived Risk of vaccines*			
			**VHS 1: n**	**mean**	**(±SD)**	**ANOVA (Stat, *p*-value. η2)**	**VHS 2: n**	**mean**	**(±SD)**	**ANOVA (Stat, *p*-value, η2)**
**Total**	5095									
**Age**										
20–29	495	9.72	485	1.509	0.689		491	2.672	0.834	
30–39	1330	26.10	1303	1.464	0.66	F = 1.486	1324	2.636	0.889	F = 2.9
40–49	1635	32.09	1614	1.522	0.723	*p* = 0.204	1626	2.642	0.868	*p* = 0.021*
50–59	1236	24.26	1217	1.492	0.722		1227	2.561	0.896	η2 = 0.002***
60+	304	5.97	299	1.463	0.601		301	2.535	0.919	
missing	95	1.86	177				126			
**Sex**										
Male	1182	23.20	1160	1.491	0.688	F = 0.006	1177	2.431	0.903	F = 69.5
Female	3809	74.76	3751	1.493	0.714	*p* = 0.937	3784	2.674	0.864	*p* <0.001**
Missing or Prefer not to answer	104	2.04	184				134			η2 = 0.01***
**Gender**										
Men	1173	23.02	1151	1.487	0.701	F = 0.461	1168	2.432	0.901	F = 34.28
Women	3794	74.47	3734	1.491	0.689	*p* = 0.63	3768	2.674	0.862	*P* <0.001**
Non-binary/Other	30	0.59	30	1.61	0.796		30	2.6	1.303	η2 = 0.01***
Missing/Prefer not to answer	98	1.92	180				129			
**Length of time in BC**										
All my life	2852	55.98	2805	1.491	0.703	F = 0.93	2833	2.618	0.862	F = 0.405
Less than 2 years	50	0.98	47	1.562	0.684	*p* = 0.445	49	2.602	0.907	*p* = 0.805
2–5 years	176	3.45	172	1.563	0.764		175	2.589	0.923	
6–10 years	148	2.90	148	1.567	0.736		147	2.704	1.03	
More than 10 years	1799	35.31	1773	1.491	0.683		1791	2.625	0.902	
Prefer not to answer/not sure/missing	70	1.37	150				100			
**Indigenous**										
Yes	150	2.94	149	1.49	0.693	F = 2.412	150	2.617	0.881	F = 1.752
No	4820	94.60	4738	1.58	0.764	*p* = 0.121	4791	2.713	0.907	*p* = 0.186
Missing/Prefer not to answer	125	2.45	208				154			
**Visible Minority**										
Yes	823	16.15	813	1.524	0.685	F = 3.063	821	2.733	0.886	F = 20.55
No	4027	79.04	3963	1.478	0.689	*p* = 0.08	4003	2.581	0.874	*p* <0.001**
Missing/Prefer not to answer	245	4.81	319				271			η2 = 0.004***
**Vaccine Knowledge Characteristics**										
**Educational area of study**										
Non-science or engineering (e.g., Arts/social sciences/Humanities/business/commerce or other)	3790	74.39	3724	1.51	0.691	F = 7.312	3762	2.664	0.88	F = 38.24
Science or Engineering	1195	23.45	1181	1.447	0.7	*p* = 0.007*	1194	2.483	0.875	*p* <0.001**
Missing/Prefer not to answer	110	2.16	190			η2 = 0.001***	139			η2 = 0.008***
**Vaccine Knowledge Scale**										
High	4501	88.34	4436	1.405	0.598	F = 636.1	4481	2.524	0.828	F = 317.2
Medium	181	3.55	180	2.041	0.683	*p* <0.001**	180	3.478	0.86	*p* <0.001**
Low	140	2.75	137	3.177	0.892	η2 = 0.21***	139	3.989	0.708	η2 = 0.12 ***
Missing	273	5.36	342				295			
**Information sources**										
**Public Health**										
Yes	3971	77.94	3912	1.452	0.647	F = 89.11	3949	2.587	0.858	F = 36.52
No	1123	22.04	1092	1.677	0.849	*p* <0.001**	1107	2.768	0.961	*p* <0.001**
						η2 = 0.02 ***				η2 = 0.007***
**Government Websites**										
Yes	2846	55.86	2805	1.46	0.688	F = 22.2	2831	2.575	0.884	F = 21.49
No	2249	44.14	2199	1.554	0.718	*p* <0.001**	2225	2.691	0.882	*p* <0.001**
						η2 = 0.004***				η2 = 0.004***
**Professional organizations (Union, School Board)**										
Yes	2070	40.63	2042	1.464	0.649	F = 9.826	2063	2.583	0.865	F = 8.206
No	3025	59.37	2962	1.527	0.736	*p* = 0.002*	2993	2.656	0.897	*p* = 0.004*
						η2 = 0.002***				η2 = 0.002***
**Health Care provider**										
Yes	2918	57.27	2881	1.457	0.653	F = 26.35	2905	2.594	0.87	F = 8.923
No	2177	42.73	2123	1.56	0.76	*p* <0.001**	2151	2.669	0.903	*p* = 0.003*
						η2 = 0.005***				η2 = 0.002***
**Traditional news sources (TV, Radio, Newspaper)**										
Yes	1484	29.13	1459	1.503	0.69	F = 0.024	1478	2.601	0.891	F = 1.685
No	3611	70.87	3545	1.5	0.708	*p* = 0.877	3578	2.637	0.881	*p* = 0.194
**Friends or colleagues**										
Yes	1109	21.77	1088	1.574	0.746	F = 15.09	1105	2.715	0.85	F = 14.23
No	3986	78.23	3916	1.481	0.689	*p* <0.001**	3951	2.601	0.893	*p* <0.001**
						η2 = 0.003***				η2 = 0.003***
**Social Media (Facebook, TikTok, Twitter, Snapchat)**										
Yes	151	2.96	148	1.608	0.677	F = 3.541	150	2.86	0.854	F = 10.8
No	4944	97.04	4856	1.498	0.703	*p* = 0.06	4906	2.619	0.885	*p* = 0.001*
										η2 = 0.002***
Prior Vaccine Behaviour										
**Up to date on all recommended vaccines**										
Yes	4475	87.83	4405	1.454	0.646	F = 70.44	4448	2.579	0.858	F = 39.94
No	98	1.92	92	2.307	1.255	*p* <0.001**	97	3.278	1.003	*p* <0.001**
Partially	256	5.02	255	1.657	0.828	η2 = 0.04***	254	2.972	0.944	η2 = 0.02***
Don’t know	245	4.81	240	1.811	0.853		244	2.826	1.014	
Prefer not to answer	21	0.41	103							
**Ever delayed or refused any vaccines**										
Yes	455	8.93	445	1.966	0.927	F = 147.2	453	3.318	0.901	F = 187.9
No	4536	89.03	4464	1.442	0.624	*p* <0.001**	4509	2.543	0.847	*p* <0.001**
Don’t know	72	1.41	71	2.076	0.973	η2 = 0.6***	70	3.221	0.815	η2 = 0.07***
Prefer not to answer	32	0.63	115				63			
**If you are a parent is your child up to date on all recommended vaccines**										
Yes	3125	61.33	3080	1.447	0.659	F = 132.9	3109	2.588	0.856	F = 58.31
No	76	1.49	73	2.902	1.244	*p* <0.001**	73	3.616	1.053	*p* <0.001**
Partially	137	2.69	133	1.974	0.758	η2 = 0.11***	135	3.252	0.803	η2 = 0.05***
Don’t know	16	0.31	16	1.929	0.747		16	2.844	0.87	
Missing/Prefer not to answer/not a parent	1741	34.17	1741				1762			
(Not a parent = 1683)										
**If you are a parent, have you ever delayed or refused a vaccine for your child**										
Yes (& don’t know)	489	9.60	477	1.999	0.867	F = 284.6	486	3.27	0.883	F = 317.4
No	2859	56.11	2820	1.42	0.659	*p* <0.001**	2842	2.532	0.836	*p* <0.001**
Missing, Prefer not to answer, or not a parent	1747	34.29	1798			η2 = 0.08***	1767			η2 = 0.09***

VHS: vaccine hesitancy scale; SD: standard deviation; ANOVA: analysis of variance; stat: F-statistic.

*p<0.05

**p<0.001

***η2 eta squared for the effect size was estimated for variables that reached significance (p<0.05) in ANOVA

The majority (87.8%) reported being up to date on all vaccines, and 8.9% reported having delayed or refused a vaccine in the past for themselves. For those who identified as a parent or guardian, 93.2% reported their child(ren) being up to date on all vaccines, and 9.6% reported having delayed or refused a vaccine in the past for their child.

Based on the 5-item general vaccine knowledge scale, 88.3% scored as having high vaccine knowledge, and most reported using reliable sources of information about vaccines, including public health (77.9%), health care providers (57.3%) and government websites (55.9%). In addition, 40.6% reported getting immunization information from a professional organization, like the local school board or teachers union, 29.1% from traditional news sources (TV, radio, print media), 21.7% from friends and colleagues, and few respondents reported getting immunization information from social media (3.0%).

**Fig 1:**
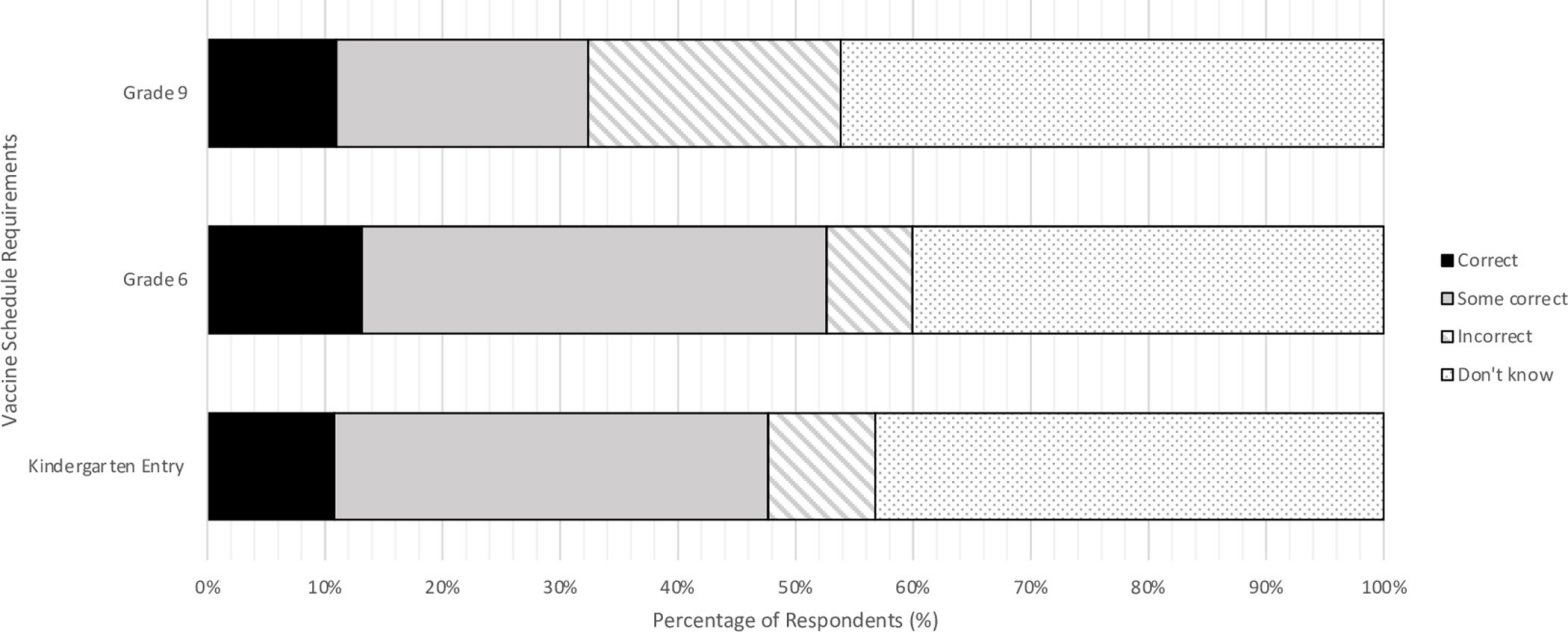
Proportion of respondents who were able to identify the recommended vaccine schedules for the school-aged immunization programs.

Knowledge of the vaccine schedule for school-age children was low with almost half of the respondents incorrectly identifying, or reporting not knowing, the vaccine schedule for kindergarten, grade 6, or grade 9 (Fig 1). Knowledge about the mature minor consent process was also low with 66.7% reporting no knowledge about this policy, and 71.8% reporting incorrectly that parental consent was always required for immunizing children 18 years or younger (Table 2).

**Table 2:** Summary of respondents’ perceived role in the school-based immunization program, by experience in grade 6 or 9 in last 5 years.

Perceived Role in the School-Based Program	Total N = 5095	Taught Grade 6 or 9 in the past 5 years n = 2553		Has NOT taught Grade 6 or 9 in the past 5 years n = 2352		
I have a clear understanding of my role in the BC school-based immunization program	n (%)	n	%	n	%	*p*-value
Agree	1437 (28.2%)	862	33.76	534	22.7	<0.0001*
Neutral	1927 (37.8%)	910	35.64	949	40.35	
Disagree	1604 (31.5%)	747	29.26	806	34.27	
Missing	127 (2.5%)	34	1.332	63	2.679	
**My role is to distribute and collect consent forms**						
Agree	2303 (45.2%)	1356	53.11	880	37.41	<0.0001
Neutral	1350 (26.5%)	557	21.82	744	31.63	
Disagree	1349 (26.5%)	622	24.36	673	28.61	
Missing	93 (1.8%)	18	0.705	55	2.338	
**My role is to manage student flow**						
Agree	1630 (32.0%)	949	37.17	635	27	<0.0001*
Neutral	1545 (30.3%)	661	25.89	823	34.99	
Disagree	1815 (35.6%)	922	36.11	834	35.46	
Missing	105 (2.1%)	21	0.823	60	2.551	
**My role is to inform students about logistics**						
Agree	2327 (45.7%)	1308	51.23	953	40.52	<0.0001*
Neutral	1390 (27.3%)	600	23.5	739	31.42	
Disagree	1259 (24.7%)	618	24.21	595	25.3	
Missing	119 (2.3%)	27	1.058	65	2.764	
**My role is to inform students about the vaccines being administered**						
Agree	1607 (31.5%)	870	34.08	681	28.95	<0.0001*
Neutral	1525 (29.9%)	690	27.03	786	33.42	
Disagree	1829 (35.9%)	962	37.68	811	34.48	
Missing	134 (2.6%)	31	1.214	74	3.146	
**My role is to inform parents about logistics**						
Agree	1230 (24.1%)	538	21.07	650	27.64	<0.0001*
Neutral	1345 (26.4%)	569	22.29	723	30.74	
Disagree	2394 (47.0%)	1414	55.39	913	38.82	
Missing	126 (2.5%)	32	1.253	66	2.806	
**My role is to inform parents about the vaccines being administered**						
Agree	834 (16.4%)	361	14.14	439	18.66	<0.0001*
Neutral	1297 (25.5%)	530	20.76	713	30.31	
Disagree	2838 (55.7%)	1629	63.81	1137	48.34	
Missing	126 (2.5%)	33	1.293	63	2.679	

* Significant at *p*<0.0001

### Vaccine confidence

**Fig 2:**
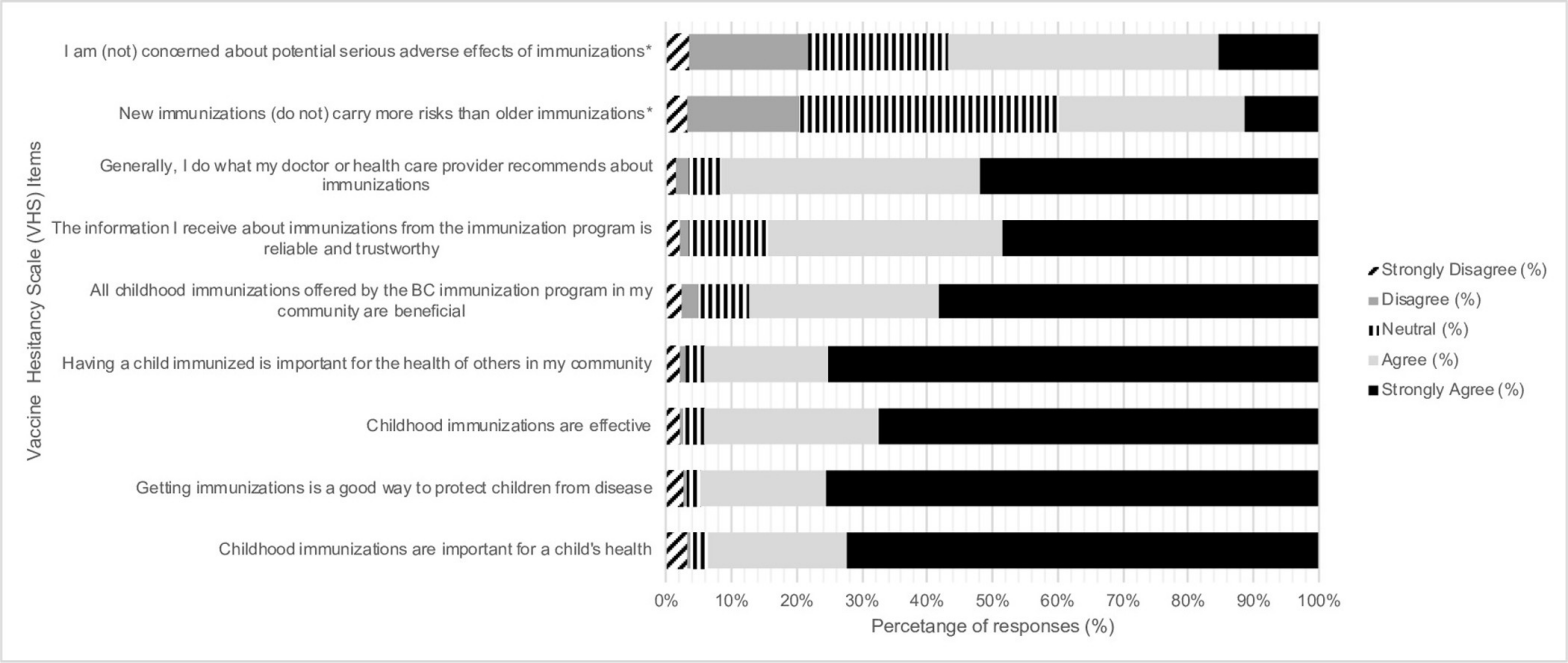
Distribution of responses to the 9 -items in the Vaccine Hesitancy Scale (VHS) for respondents (June—Nov 2020). * Indicates items that were reverse-scored, with the (text in parentheses) the edited wording of the original survey item.

Overall general vaccine confidence was high, based on the low mean scores of the VHS and the overall distribution of the 9-items of the VHS (Fig 2). The mean score on the 7-item sub-scale ‘*lack of confidence in vaccines’* was 1.5 (SD 0.7), and the mean score on the 2-item *‘perceived risk of vaccines’* sub-scale was 2.6 (SD 0.88).

#### ‘Lack of confidence in vaccines’ sub-scale

There were significant differences in general vaccine confidence based on the *‘lack of confidence in vaccines’* subscale by sociodemographic characteristics, vaccine knowledge, and prior vaccine behaviour (Table 1). Higher vaccine confidence was statistically associated with having an educational background in a scientific discipline, being up to date on all recommended vaccines (for yourself or a child), never having delayed or refused a vaccine (for yourself or a child), having high general vaccine knowledge, and using reliable sources of information about vaccines, as opposed to friends and colleagues. The effect size, or strength of association, with vaccine confidence was largest in those with higher general vaccine knowledge (η^2^ = 0.21), never delayed or refused a vaccine (η^2^ = 0.6), being up to date on vaccines (η^2^ = 0.04). For those that reported being parents/guardian, the largest effects sizes were measured in those that reported their child being up to date on vaccines (η^2^ = 0.11) and never delayed a vaccine for their child (η^2^ = 0.08) (Table 1). The effect size was small (η^2^<0.02) for all other statistically significant predictors. There was no significant association found between age, sex, gender, identifying as a visible minority, Indigeneity, or length of time residing in BC and ‘lack of confidence’ in vaccines.

#### ‘Perceived risk of vaccines sub-scale’

There were significant differences in general vaccine confidence based on the ‘*perceived risk of vaccines’* sub-scale by sociodemographic characteristics, vaccine knowledge, and prior vaccine behaviour (Table 1). Higher vaccine confidence was statistically associated with older age, male sex, identifying as a man, and not identifying as a visible minority. Lower perceived risk of vaccines was associated with having an educational background in a scientific discipline, having high general vaccine knowledge, and using reliable sources of information about vaccines. Those that reported using friends and colleagues and social media for information sources about vaccines had higher perceived risk of vaccines (Table 1). Prior vaccine behaviour was associated lower perceived risk, with those being up to date on all recommended vaccines (for yourself or a child) or never having delayed or refused a vaccine (for yourself or a child) reporting lower ‘perceived risk of vaccines’.The largest effect size, or strength of association, for vaccine confidence related to perceived risk was for general vaccine knowledge (η^2^ = 0.12), never delayed or refused a vaccine (η^2^ = 0.07), and for those that reported being parents/guardian: child being up to date on vaccines (η^2^ = 0.05) and ever delayed a vaccine for their child (η^2^ = 0.09) (Table 1). The effect size was small (η^2^<0.02) for all other statistically significant predictors. There was no significant association with perceived risk of vaccines and Indigeneity or length of time residing in BC.

### Perceived role in the school-based immunization program

A significant difference in opinion existed on the perceived role in the school-based program based on if a participant reported having taught grade 6 or 9 in the last 5 years (Table 2). Of the teachers who reported having taught grade 6 or 9 in the last 5 years, 33% reported having a clear idea of their role in the school-based program compared to only 22% of those who have not taught grade 6 or 9 (p<0.0001) (Table 3). A significantly higher proportion of teachers who had taught grade 6 or 9 in the last 5 years responded that their role in the school-based immunization program was to collect consent forms, inform students about logistics, and manage student flow during the school immunization clinics. In regard to communicating about vaccines to students and parents, 34% of teachers who had taught grade 6 or 9 in the last 5 years reported that their role was to inform students about the vaccines being administered, while only 14% agreed their role was to inform parents about the vaccines being administered.

**Table 3:** Summary of respondents’ perceived role in the school-based immunization program and opinion on sharing beliefs and measured vaccine hesitancy.

		VHS Factor 1: *Lack of confidence in vaccines*				VHS Factor 2: *Perceived Risk of vaccines*			
Sharing Personal Views about Immunizations	Total n (%)	VHS1: n	mean	(±SD)	ANOVA (Stat, d.f., p-value)	VHS2: n	mean	(±SD)	ANOVA (Stat, d.f., p-value,)
**It is appropriate for me to share my personal opinions about vaccines with students**									
Agree	1042 (20.5%)	1025	1.337	0.628	F = 42.01	1035	2.351	0.86	F = 71.59
Neutral	1213 (23.8%)	1197	1.479	0.67	*p* <0.0001*	1208	2.609	0.835	*p*<0.0001*
Disagree	2763 (54.2%)	2715	1.569	0.729	n2 = 0.02**	2748	2.732	0.895	n2 = 0.03**
Missing	77 (1.5%)	158							
**It is appropriate for me to share my personal opinions about vaccines with parents**									
Agree	900 (17.7%)	886	1.363	0.658	F = 24.93	896	2.353	0.87	F = 56.83
Neutral	1284 (25.2%)	1271	1.482	0.669	*p* <0.0001*	1278	2.619	0.849	*p*<0.0001*
Disagree	2844 (55.8%)	2789	1.55	0.722	n2 = 0.01**	2827	2.711	0.889	n2 = 0.02**
Missing	67 (1.3%)								

VHS: vaccine hesitancy scale; SD: standard deviation; ANOVA: analysis of variance; stat: F-statistic.

*p<0.0001

**η2 eta squared for the effect size was estimated for variables that reached significance (p<0.05) in ANOVA

### Sharing vaccine beliefs with students and parents

Half of teachers did not think it was appropriate to share personal opinions on vaccines with students (54%) compared to 20.5% who felt it was appropriate, which was similar to their opinions with sharing personal beliefs with parents (Table 2). Respondents who agreed that it was appropriate for them to share their personal opinion on vaccines with students and/or parents had higher vaccine confidence based on both VHS sub-scales (Table 3).

## Discussion

Overall, public-school teachers in BC had high general vaccine confidence. Vaccine hesitancy is complex and there is a need to measure and characterize over time within populations, to be able to best address and monitor the potential impacts of vaccine hesitancy and levels of vaccine confidence [16]. The VHS sub-scales identified that among teachers, vaccine hesitancy was related more to the perceived risk, or safety, of vaccines, compared to confidence in the effectiveness of vaccines. Perceived risk of vaccines as the driving factor for vaccine hesitancy has been previously observed in studies of parents and vaccine hesitancy, specifically related to adolescent vaccines [16, 23]. In comparison to other population-based studies that have used the VHS as a metric, teachers had overall lower mean scores, indicating overall higher general vaccine confidence [16, 18].

The effect size, or strength of association, between participant sociodemographic characteristics and general vaccine confidence were overall small (η^2^< 0.02), which is consistent with other population-based studies exploring vaccine confidence using the VHS [16, 18, 19]. However, the sociodemographic characteristics associated with the sub-scales scores varied, with few observed significant associations with the ‘lack of confidence’ in vaccines’ based on sociodemographic factors, or group level differences. In comparison, the ‘perceived risk of vaccines’ sub-scale identified that younger teachers, those who identified as women or are female, and identify as a visible minority have higher perceived risk of vaccines. However, despite these sociodemographic differences, the effect size was small, with stronger associations related to prior vaccine behaviour and vaccine knowledge.

The strongest associations for high general vaccine confidence were reporting never having previously delayed or refused a vaccine in the past and having high general vaccine knowledge. Those who reported delaying or refusing a vaccine, or being unsure if they had delayed or refused vaccines in the past, had higher vaccine hesitancy for both VHS sub-scales compared to those who had never delayed or refused a vaccine in the past. This observed relationship between the prior vaccine behaviour in the refusal or delay of vaccination and measured vaccine confidence was indicative of the validity of the scales in assessing past vaccine hesitancy in this population.

Overall, general knowledge of vaccines was high, with >88% of those scoring 4.0/5.0 or greater on the general vaccine knowledge scale. Despite the high level of general vaccine knowledge, there was a clear incremental trend of increased vaccine confidence with increasing vaccine knowledge, with the strongest association being for the sub-scale measuring ‘*lack of confidence in vaccines*’ (η^2^ = 0.21) compared to the *‘perceived risk of vaccines’* sub-scale (η^2^ = 0.12). The observed relationship between increased vaccine knowledge and higher vaccine confidence is consistent with other studies of teachers, which found those with higher vaccine knowledge levels are more likely to be vaccinated [24, 25].

Specific knowledge of school-based immunization schedules was low among respondents, which was not surprising given explicit vaccination education is not part of their employment mandate as teachers. In addition, respondents were asked to identify individual vaccine-preventable illnesses covered by the vaccines in the age (grade) specific vaccine schedules, as opposed to the vaccine trade names or abbreviations, which may have caused some misclassification of knowledge if a respondent was more familiar with the vaccine trade names or abbreviations themselves (e.g., MMR or HPV). In regard to knowledge about the immunization program, the majority of respondents were unaware of mature minor consent and believed that parental consent is required for all vaccinations of minors. This knowledge and awareness gap in the mature minor consent policy is an opportunity for public health to engage teachers through the immunization program, which could impact vaccine acceptance among students, particularly in grade 9 (14- and 15-year-olds), for which mature minor consent is currently offered. Teachers are well situated to speak to students about mature minor consent and adolescent vaccines, particularly within the capacity of the health education curriculum, and could play a role in improving adolescent vaccine acceptance and future acceptance [26, 27].

Our population of teachers is highly educated, with all teachers in BC requiring a bachelor’s degree, in addition to their teaching qualification. We explored if the educational training background was associated with vaccine confidence and found that those with an educational background in a scientific discipline (e.g., biology, engineering) had higher vaccine confidence, for both sub-scales of the VHS. The association between formal scientific training and higher vaccine confidence underscores previous work, reporting on the role of scientific education and science literacy being associated with increased vaccination and vaccine confidence [8, 28]. In a highly educated population of teachers, the association between formal scientific training and increased vaccine confidence suggests that a public health partnership opportunity with science teachers to increase vaccine knowledge may be a successful strategy to increase vaccine confidence among teachers and students [8].

Public health is ideally situated as a reliable source of information for teachers, given that when asked about the main sources of information about immunizations, teachers reported public health as the top source, followed by health care providers and government websites. Participants also highly ranked unions and school boards as a source of information, which is an avenue worth exploring in partnership with public health to provide accurate sharable information about vaccines and vaccine-preventable diseases to teachers, to help mitigate any potential risks associated with misinformation.

Communication regarding vaccination is a shared community responsibility [29], and teachers are often an intermediary between parents/guardians/caregivers and public health officials. Subsequently, all stakeholders in the school-based immunization program, including teachers, could play an important role as a trusted source of information about immunizations. The opportunity to partner with teachers to provide immunization information is worth future exploration. We found respondents who reported that sharing information about vaccines with students and/or parents was appropriate, were on average more vaccine confident. This finding was in contrast to a recent study of American teachers, which found teachers who challenged the science of vaccines were more likely to discuss vaccines in the classroom, however, the authors noted that with directives and appropriate standards within the curriculum, teachers could be supported to have a major role in increasing vaccine literacy, and uptake among students [27].

Within the BC school-based immunization program, currently the primary role of teachers is to assist in the coordination of the requirements for public health, and includes, but may not be limited to, distribution and collection of consent forms and coordination of students on the day of the school immunization clinic. Teachers’ self-reported role in the school-based immunization program significantly differed based on if they had taught grade 6 or 9 in the last 5 years. The school-based immunization programs are delivered in grade 6 and 9, and it is not surprising that in regard to logistics, collecting consent forms, and providing student management at the immunization clinics was associated with having recently taught grades in which the school immunization program is delivered. However, overall, there was a wide distribution of the perceived role of teachers in the school immunization program. This presents an opportunity to engage with teachers on how they can support school-based immunization in both a functional role (i.e., consent forms, logistics) but also as conduits for general immunization information and program specific information around mature minor consent and what vaccines are recommended.

### Limitations

This study is not without limitations. There is the potential for non-response bias from those who oppose vaccination, or are highly vaccine hesitant, given the study was conducted by an institution that promotes vaccines. In addition, the complete case analysis may have introduced bias by systematically excluding those who oppose vaccines, if they opened the survey (e.g., clicked the link), but did not submit the survey as complete. However, we believe the bias would have been minimal, given that vaccine deniers are estimated to be a small percentage of the population (2–4%). We achieved a sample size of >5,000 respondents, which was representative of the BC teacher workforce based on sex, age, and geographical location of respondents.

A limitation of the VHS itself is that it primarily focuses around confidence in the effectiveness of vaccines, with the sub-scale regarding perceived risks comprising only 2 items. Additional work is needed to bolster the scale items, and expand the scope of the scale to address additional factors beyond confidence and risk [19, 30]. The utility of the scale is currently limited to surveillance and descriptive analysis of vaccine hesitancy in populations, and future work on the predictive validity of the scale should be explored for personal vaccine uptake or specific vaccine endorsement. The VHS wording is specific to childhood vaccines, which typically has been applied to a parental population, however, we felt it was appropriate wording given the role that teachers play in the lives of their students. However, we did modify the exact wording of the items to be towards children in general and not a respondent’s child. Other alterations in the item wording to expand the scale’s applicability has not altered the reliability of the scale in other populations [18], but we did not test these alterations in our population.

Lastly, it is important to note that this study and data collection was conducted during the first summer and fall of the COVID-19 pandemic [31]. At the time of data collection, BC was beginning to enter the second large wave of COVID-19, with daily hospitalizations rising from <1.0 per 100, 000 population at the beginning of August 2020 to 2.0 per 100,000 population by early November 2020 [32], with schools projected to be open and remained open during fall 2020. COVID-19 vaccination was not the focus on this analysis however, participants were asked about their intention to receive a future COVID-19 vaccine, and these data have been published elsewhere [31]. The COVID-19 pandemic may have influenced overall vaccine confidence, which highlights the importance of continued monitoring of vaccine confidence and hesitancy over time.

## Conclusions

Using a validated scale, we found overall teachers in BC have high confidence in vaccines and are well situated as potential partners in public health to address vaccine hesitancy. This exploratory study of a large population sample of public-school teachers highlights a number of key opportunities to engage with teachers on vaccination with the school-based immunization program. A key implication of the study is that public school teachers are well situated to be partners with public health to provide information on vaccinations and take on an active role in the school-based immunization program. In conjunction with teachers, administrators, and public health officials, key resources to support teachers should be developed, and implementation measured for both the impact on vaccine confidence amongst teachers and uptake of school-based immunizations.

## Supporting information

S1 File Survey. (DOCX)

S1 Table BC immunization schedule. (As of May 2021).
(DOCX)

## References

[1] MacDonald NE, Eskola J, Liang X, Chaudhuri M, Dube E, Gellin B, et al. Vaccine hesitancy: Definition, scope and determinants. Vaccine. 2015;33(34):4161–4. doi: 10.1016/j.vaccine.2015.04.036 25896383

[2] World Health Organization. Ten Threats to Global Health in 2019 [Internet]. 2019 [cited 2020 Dec 2]. Available from: https://www.who.int/news-room/spotlight/ten-threats-to-global-health-in-2019#:∼:text= These range from outbreaks of,change and multiple humanitarian crises.

[3] BC Centre for Disease Control. Communicable Disease Control Manual Chapter 2: Immunization Introduction [Internet]. BC Centre for Disease Control. 2018 [cited 2022 May 8]. Available from: http://www.bccdc.ca/resource-gallery/Documents/Guidelines%20and%20Forms/Guidelines%20and%20Manuals/Epid/CD%20Manual/Chapter%202%20-%20Imms/Part_1_Schedules.pdf

[4] ImmunizeBC BC Centre for Disease Control. The Infants Act, Mature Minor Consent and Immunization [Internet]. HealthLinkBC File Number: 119. 2021 [cited 2021 Sep 21]. Available from: https://www.healthlinkbc.ca/healthlinkbc-files/infants-act-mature-minor-consent-and-immunization

[5] BC Centre for Disease Control. Immunization Coverage in Grade 9 Students 2011–2020 [Internet]. 2019 [cited 2022 May 8]. Available from: http://www.bccdc.ca/resource-gallery/Documents/Statistics%20and%20Research/Statistics%20and%20Reports/Immunization/Coverage/Grade%209%20Coverage%20Results.pdf

[6] Dubé E, Gagnon D, Clément P, Bettinger JA, Comeau JL, Deeks S, et al. Challenges and opportunities of school-based HPV vaccination in Canada. Hum Vaccin Immunother. 2019;15(7–8):1650–5. doi: 10.1080/21645515.2018.1564440 30633622 PMC6746476

[7] Gkentzi D, Benetatou E, Karatza A, Marangos M, Varvarigou A, Dimitriou G. Knowledge and attitudes of school teachers on vaccination in Greece. Infect Chemother. 2021;53(2):364–7. doi: 10.3947/ic.2020.0153 34216129 PMC8258291

[8] Frayon S. New Caledonian biology teachers’ opinions about vaccination: Preliminary findings. Health Educ J. 2020;79(5):594–606.

[9] Harris PA, Taylor R, Thielke R, Payne J, Gonzalez N, Conde JG. Research electronic data capture (REDCap)-A metadata-driven methodology and workflow process for providing translational research informatics support. J Biomed Inform. 2009;42(2):377–81. doi: 10.1016/j.jbi.2008.08.010 18929686 PMC2700030

[10] The American Association for Public Opinion Research. Standard Definitions Final Dispositions of Case Codes and Outcome Rates for Surveys. 9th edition. 2016.

[11] Ministry of Education of British Columbia. British Columbia Public School Teacher Statistics [Internet]. 2018. Available from: https://catalogue.data.gov.bc.ca/dataset/76a16627-90a2-4d94-a8d0-dfe2b7d0a87f

[12] British Columbia Teachers’ Federation (BCFT). British Columbia Teachers’ Federation [Internet]. 2020 [cited 2020 Dec 9]. Available from: https://www.bctf.ca/

[13] British Columbia Teachers’ Federation. BCTF Research Report—Teachers in British Columbia: A feminized workforce. 2018.

[14] Statistics Canada. Dictionary, Census of Population, 2016 Visible minority [Internet]. Catalogue no. 98-301-X. 2017. Available from: https://www12.statcan.gc.ca/census-recensement/2016/ref/dict/pop127-eng.cfm

[15] Zingg A, Siegrist M. Measuring people’s knowledge about vaccination: Developing a one-dimensional scale. Vaccine. 2012;30(25):3771–7. doi: 10.1016/j.vaccine.2012.03.014 22445808

[16] Shapiro GK, Tatar O, Dube E, Amsel R, Knauper B, Naz A, et al. The vaccine hesitancy scale: Psychometric properties and validation. Vaccine. 2018;36(5):660–7. doi: 10.1016/j.vaccine.2017.12.043 29289384

[17] Larson HJ, Jarrett C, Schulz WS, Chaudhuri M, Zhou Y, Dube E, et al. Measuring vaccine hesitancy: The development of a survey tool. Vaccine. 2015;33(34):4165–75. doi: 10.1016/j.vaccine.2015.04.037 25896384

[18] Luyten J, Bruyneel L, van Hoek AJ. Assessing vaccine hesitancy in the UK population using a generalized vaccine hesitancy survey instrument. Vaccine. 2019;37(18):2494–501. doi: 10.1016/j.vaccine.2019.03.041 30940484

[19] Domek GJ, O’Leary ST, Bull S, Bronsert M, Contreras-Roldan IL, Ventura GAB, et al. Measuring vaccine hesitancy: Field testing the WHO SAGE Working Group on Vaccine Hesitancy survey tool in Guatemala Gretchen. Vaccine. 2018;36(35):5273–81. 30061026 10.1016/j.vaccine.2018.07.046 PMC6145454

[20] Wagner AL, Masters NB, Domek GJ, Mathew JL, Sun X, Asturias EJ, et al. Comparisons of vaccine hesitancy across five low- and middle-income countries. Vaccines (Basel). 2019;7(4):1–11. doi: 10.3390/vaccines7040155 31635270 PMC6963484

[21] R Core Team. The R Project for Statistical Computing: v.4.0.1. Vienna, Austria: R Foundation for Statistical Computing; 2020.

[22] Government of British Columbia. British Columbia Population Estimates [Internet]. BC Stats. 2020 [cited 2020 Dec 10]. Available from: https://www2.gov.bc.ca/gov/content/data/statistics/people-population-community/population/population-estimates

[23] Helmkamp LJ, Szilagyi PG, Zimet G, Saville AW, Gurfinkel D, Albertin C, et al. A validated modification of the vaccine hesitancy scale for childhood, influenza and HPV vaccines. Vaccine. 2021;39(13):1831–9. doi: 10.1016/j.vaccine.2021.02.039 33676784 PMC13325669

[24] Ganczak M, Kalinowski P, Drozd-Dąbrowska M, Biesiada D, Dubiel P, Topczewska K, et al. School life and influenza immunization: A cross-sectional study on vaccination coverage and influencing determinants among Polish teachers. Vaccine. 2020;38(34):5548–55. doi: 10.1016/j.vaccine.2019.10.067 31706813

[25] Macintosh J, Luthy KE, Beckstrand RL, Eden LM, Orton J. Vaccination perceptions of school employees in a rural school district. Vaccine. 2014;32(37):4766–71. doi: 10.1016/j.vaccine.2014.06.029 25024111

[26] Maisonneuve AR, Witteman HO, Brehaut Jamie, Dub E, Wilson K. Educating children and adolescents about vaccines: a review of current literature. Expert Rev Vaccines. 2018;17(4):311–21. doi: 10.1080/14760584.2018.1456921 29569498

[27] Plutzer E, Warner SB. A potential new front in health communication to encourage vaccination: Health education teachers. Vaccine. 2021;39(33):4671–7. doi: 10.1016/j.vaccine.2021.06.050 34215451

[28] de Figueiredo A, Simas C, Karafillakis E, Paterson P, Larson HJ. Mapping global trends in vaccine confidence and investigating barriers to vaccine uptake: a large-scale retrospective temporal modelling study. The Lancet. 2020;396(10255):898–908. doi: 10.1016/S0140-6736(20)31558-0 32919524 PMC7607345

[29] Biasio LR, Corsello G, Costantino C, Fara GM, Giammanco G, Signorelli C, et al. Communication about vaccination: A shared responsibility. Hum Vaccin Immunother. 2016;12(11):2984–7. doi: 10.1080/21645515.2016.1198456 27458874 PMC5137540

[30] Betsch C, Schmid P, Heinemeier D, Korn L, Holtmann C, Böhm R. Beyond confidence: Development of a measure assessing the 5C psychological antecedents of vaccination. Vol. 13, PLoS ONE. 2018. 1–32 p. doi: 10.1371/journal.pone.0208601 30532274 PMC6285469

[31] Racey CS, Donken R, Porter I, Albert A, Bettinger JA, Mark J, et al. Intentions of public school teachers in British Columbia, Canada to receive a COVID-19 vaccine. Vaccine X. 2021;8:100106. doi: 10.1016/j.jvacx.2021.100106 34222854 PMC8240436

[32] BCCDC COVID-19 Epidemiology App [Internet]. BC Centre for Disease Control. [cited 2022 Oct 13]. Available from: https://bccdc.shinyapps.io/covid19_global_epi_app/

