# Supplementary material for: Characterization of vaccine confidence among teachers in British Columbia, Canada: A population-based survey
Source: PLoS One. 2023 Jul 12;18(7):e0288107. doi: 10.1371/journal.pone.0288107 (PMC10337953; doi:10.1371/journal.pone.0288107)
Supplement: S1 File — (DOCX) [file pone.0288107.s001.docx]

**Supplemental Material – Survey**

**Section - Your Immunization experience**

*To begin, we would now like to learn more about* ***your personal experience*** *with immunizations, and some of the reasons why, or why not, you have chosen to get immunized.*

**Q1: To the best of your knowledge, are you up to date on all recommended immunizations?**

□ Yes

□ No

□ Partially

□ Don’t know

□ Prefer not to answer

**Q2: Have you ever delayed or refused any immunizations for a non-medical reason?**

- Yes, I’ve delayed Immunizations in the past
- Yes, I’ve refused immunizations in the past
- No
- Don’t know
- Prefer not to answer

**Q3: If you are a parent/guardian/caregiver of a child, to the best of your knowledge are your children up to date on all recommended immunizations?**

□ Not applicable (I am not a parent/guardian/caregiver of a child)

□ Yes

□ No

□ Partially

□ Don’t know

□ Prefer not to answe**r**

**Q4: [SKIP if Q3 = Not applicable] If you are a parent/guardian/caregiver of a child, have you ever delayed or refused immunizations for a child for a non-medical reason?**

Not applicable (I am not a parent/guardian/caregiver of a child)

- Yes, I’ve delayed immunizations in the past
- Yes, I’ve refused immunizations in the past
- No
- Don’t know

Prefer not to answer

**Q5a [skip pattern - Q1: Yes/Partial/Don’t know] Could you please tell us your main reasons why you have said yes to immunizations for yourself in the past?** (the rank your top three reasons)

□ Immunizations are important for my health

□ My health care provider or public health nurse recommended I get immunized

□ I get immunized because it protects the health of others in my community

□ Immunizations protect me against diseases

□ Immunizations stop the spread of diseases

□ Immunization saves money

□ Immunization protects future generations

□ Immunizations are the best way to protect children from preventable diseases

□ Immunization helps me maintain my lifestyle

□ I had to get immunized for work or school

□ Other, please specify _________

**Q5b [skip pattern, Q1: No/partial/don’t know or Q2 yes to delay or refuse] Could you please tell us your main reasons for why you have ever said no to an immunization for yourself in the past?**

□ I had difficulty getting an appointment to get immunized

□I was worried about side effects from an immunization

□The immunization was too new

□ The risk of disease is small

□ A health care provider advised me not to get immunized.

Please specify health care provider type:

□My church/religion advised me not to get immunized

□ I don’t have enough information to decide

□ I have a medical condition that prevents me from getting immunized

□ I don’t believe the immunizations work

□ I do not like needles

□ I didn’t get sick before so I don’t need it now

□ I worry that immunizations will make me sick with the disease

□ Other, please specify _________

**Q6: In general, I consider my knowledge of immunizations to be…**

- 5 - Excellent
- 4 - Good
- 3 - Neutral
- 2 - Poor
- 1 - Very poor

**Section – Attitude towards vaccination/vaccine confidence**

[**Section Instructions:]** *The next section will ask you about immunizations in general.*

*For the following statements, please indicate on a scale of 1 – 5, with 1 being strongly disagree and 5 being strongly agree:*

**Q7: Childhood immunizations are important for a child’s health**

[*5 point scales 1 strongly disagree, 3 neutral and 5 strongly agree]*

**Q8: Getting immunizations is a good way to protect children from disease**

[*5 point scales 1 strongly disagree, 3 neutral and 5 strongly agree]*

**Q9: Childhood immunizations are effective**

[*5 point scales 1 strongly disagree, 3 neutral and 5 strongly agree]*

**Q10: Having a child immunized is important for the health of others in my community**

[*5 point scales 1 strongly disagree, 3 neutral and 5 strongly agree]*

**Q11: All childhood immunizations offered by the BC immunization program in my community are beneficial** [*5 point scales 1 strongly disagree, 3 neutral and 5 strongly agree]*

**Q12: The information I receive about immunizations from the immunization program is reliable and trustworthy** [*5 point scales 1 strongly disagree, 3 neutral and 5 strongly agree]*

**Q13: Generally, I do what my doctor or health care provider recommends about immunizations** [*5 point scales 1 strongly disagree, 3 neutral and 5 strongly agree]*

**Q14: New immunizations carry more risks than older immunizations**

[*5 point scales 1 strongly disagree, 3 neutral and 5 strongly agree]*

**Q15: I am concerned about potential serious adverse effects of immunizations**

[*5 point scales 1 strongly disagree, 3 neutral and 5 strongly agree]*

**Q16: Children do not need immunizations for diseases that are not common anymore.**

[*5 point scales 1 strongly disagree, 3 neutral and 5 strongly agree]*

**Q17: The risk associated with diseases is larger than the risk associated with immunizations**

[*5 point scales 1 strongly disagree, 3 neutral and 5 strongly agree]*

**Q18: Most parents I know immunize their children**

[*5 point scales 1 strongly disagree, 3 neutral and 5 strongly agree]*

**Q19: School is the ideal environment to offer immunizations**

[*5 point scales 1 strongly disagree, 3 neutral and 5 strongly agree]*

**Immunization Knowledge**

*The next questions will ask you about your knowledge of immunizations and the school-based immunization program. There are no right or wrong answers, please answer to the best of your knowledge.*

**Q20: Please check the immunizations that are given at school entry in BC (Kindergarten/Grade 1)? (please select all that apply)**

None

Diphtheria

Flu (influenza)

*Haemophilus influenzae* type b (Hib)

Hepatitis A

Hepatitis B

Human papillomavirus (HPV)

Measles

Mumps

Meningococcal disease

Pertussis (whooping cough)

Pneumococcal disease

Polio

Rotavirus

Rubella

Tetanus

Varicella (Chicken Pox)

Other

Don’t know

**Q21: Please check the immunizations that are given in grade 6? (please select all that apply)**

None

Diphtheria

Flu (influenza)

*Haemophilus influenzae* type b (Hib)

Hepatitis A

Hepatitis B

Human papillomavirus (HPV)

Measles

Mumps

Meningococcal disease

Pertussis (whooping cough)

Pneumococcal disease

Polio

Rotavirus

Rubella

Tetanus

Varicella (Chicken Pox)

Other

Don’t know

**Q22: Please check the immunizations that are given in grade 9? (please select all that apply)**

None

Diphtheria

Flu (influenza)

*Haemophilus influenzae* type b (Hib)

Hepatitis A

Hepatitis B

Human papillomavirus (HPV)

Measles

Mumps

Meningococcal disease

Pertussis (whooping cough)

Pneumococcal disease

Polio

Rotavirus

Rubella

Tetanus

Varicella (Chicken Pox)

Other

Don’t know

**Q24: Please indicate if you believe the following statements are TRUE or FALSE.**

| 1. In BC, once a student is eligible for a vaccine on the routine immunization schedule they are always eligible to receive that immunization, except for the HPV vaccine | **T / F** |
| --- | --- |
| 1. All immunizations offered in the BC school-based program are free of charge | **T/ F** |
| 1. In BC, children under the age of 19 always require parental consent for immunization | **T / F** |
|  |  |
| 1. Immunizations are effective at preventing diseases | **T/F** |
| 1. All immunizations provide lifelong immunity | **T/F** |
| 1. It is safer to get immunized than to get a disease | **T/F** |
| 1. The majority of side effects from immunizations are mild, and last for only a day or two. | **T/F** |
| 1. Ingredients in immunizations are safe for children and adults. | **T/F** |
| 1. Multiple immunizations at one time will not overload a child’s immunize system | **T/F** |
| 1. Chickenpox in children can cause severe disease, including death. | **T/F** |
| 1. Immunizations only protect the person who got the vaccine. | **F/F** |
| 1. The Hepatitis B vaccine and the HPV vaccine can prevent cancer | **T/F** |

**Q25: Have you heard of mature minor consent?**

□ Yes

□ No

□ Prefer not to answer

Looking for information on up to date information about a vaccine, vaccine preventable diseases, and immunization schedules: CHECK OUT 🡪 <https://immunizebc.ca/>

**Section - School**

*In this set of questions, we would like to learn more about you and the school you are teaching at for statistical purposes. We would like to get a better idea of where you teach and your school composition, please remember your responses are not identifiable.*

**Q26: What school district are you currently working in? (if you work in more than one school district, please choose the districts where you work most of the time):**

□ 5 Southeast Kootenay

□ 6 Rocky Mountain

□ 8 Kootenay Lake

□ 10 Arrow Lakes

□ 19 Revelstoke

□ 20 Kootenay-Columbia

□ 22 Vernon

□ 23 Central Okanagan

□ 27 Cariboo-Chilcotin

□ 28 Quesnel

□ 33 Chilliwack

□ 34 Abbotsford

□ 35 Langley

□ 36 Surrey

□ 37 Delta

□ 38 Richmond

□ 39 Vancouver

□ 40 New Westminster

□ 41 Burnaby

□ 42 Maple Ridge-Pitt Meadows

□ 43 Coquitlam

□ 44 North Vancouver

□ 45 West Vancouver

□ 46 Sunshine Coast

□ 47 Powell River

□ 48 Howe Sound

□ 49 Central Coast

□ 50 Haida Gwaii/Queen Charlotte

□ 51 Boundary

□ 52 Prince Rupert

□ 53 Okanagan Similkameen

□ 54 Bulkley Valley

□ 57 Prince George

□ 58 Nicola-Similkameen

□ 59 Peace River South

□ 60 Peace River North

□ 61 Greater Victoria

□ 62 Sooke

□ 63 Saanich

□ 64 Gulf Islands

□ 67 Okanagan Skaha

□ 68 Nanaimo-Ladysmith

□ 69 Qualicum

□ 70 Alberni

□ 71 Comox Valley

□ 72 Campbell River

□ 73 Kamloops/Thompson

□ 74 Gold Trail

□ 75 Mission

□ 78 Fraser-Cascade

□ 79 Cowichan Valley

□ 81 Fort Nelson

□ 82 Coast Mountains

□ 83 North Okanagan-Shuswap

□ 84 Vancouver Island West

□ 85 Vancouver Island North

□ 87 Stikine

□ 91 Nechako Lakes

□ 92 Nisga'a

**Q27: What is the postal code of your school that you currently work? __________________**

**Section - Information and resources**

*In the next series of questions, we would like to learn more about what sources of information you use regarding immunizations and what information, if any, should be a part of the educational curriculum.*

**Q28: Where do you receive your information on immunizations? Please select your top 5 sources of information.**

Public health nurses or officials

Health Authority website

Government websites (e.g. Ministry of Health)

HealthLinkFilesBC

ImmunizeBC or BCCDC

School District

BC Teachers Federation

News (e.g. television or print media)

News websites

Facebook

Instagram

TikTok

Twitter

YouTube

Snapchat

Tumblr

On blogging sites

Healthcare providers

Colleagues and/or friends

Other websites, please specify _______

Other, please specify _______

Prefer not to answer

**Q29: Where do you receive your information about the BC school-based immunization program? (Please select all that apply)**

- - Public health nurses
  - Government Websites
  - ImmunizeBC
  - School District
  - Other
  - I have not received any information about the school-based immunization program

**Q30: What do you think your students should know about immunizations and the BC school-based immunization program? (Please select all that apply)**

□ How immunizations work

□ How immunizations protect against disease

□ Safety of immunizations

□ Disease information

□ The recommended immunization schedule

□ Rules and regulations surrounding children’s ability to make their own decisions regarding immunization

□ Students **do not** need to know about immunizations or the BC school-based immunization program, as their parents make that decision for them.

□ Other

□ Prefer not to answer

**Q31: Would you like to receive more information from public health regarding the BC school-based immunization program?**

□ Yes

□ No

□ Prefer not to answer

**Q31a: If so, what information would you like to receive? (please select all that apply)**

□ How immunizations work

□ How immunizations protect against disease

□ Safety of immunizations

□ Disease information

□ The recommended immunization schedule

□ Rules and regulations surrounding children’s ability to make their own decisions regarding immunization

□ How to communicate with students about immunizations

□ How to communicate with parents about immunizations

□ Other

□ Prefer not to answer

**Q31b: If so, how would you prefer to receive this information? (Select all that apply)**

□ Online website/course

□ A workshop with other teachers

□ An information package/leaflet

□ One on one conversation with a public health nurse

□ Other

□ Prefer not to answer

**Section: Practices**

*We would now like to ask you about your experience and role in the BC school-based vaccination program and KidsBoostImmunity.*

**KidsBoostImmunity is an online teaching resource linked with science, social studies, and other curriculums. By completing quizzes, students have the opportunity to earn vaccines for children through UNICEF Canada. The more a student learns, the more vaccines they earn for children in need through UNICEF. KidsBoostImmunity is a non-profit Canadian education initiative made possible through a partnership between Public Health Agency of Canada, Public Health Associations of British Columbia and BC Centre for Disease Control.** <https://kidsboostimmunity.com/>

**Q32: Are you familiar with KidsBoostImmunity?**

□ Yes 🡪 Q32a

□ No

□ Prefer not to answer

**Q32a: Do you use KidsBoostImmunity in your classroom?**

□ Yes 🡪 Q32b

□ No

□ Prefer not to answer

**Q32b: Is there anything you would like to share with us about KidsBoostImmunity or your experience with KidsBoostImmunity 🡪** Open text box

**Q33: Is immunization education part of the regular curriculum for your grade?**

□ Yes

□ No

□ No, but I include in anyway

□ Not sure

□ Prefer not to answer

**Q34: Do you think information on vaccine preventable diseases (e.g. polio, mumps, chicken pox, measles, influenza) should be part of the curriculum?**

□ Yes, but I don’t have enough background 🡪 Go to Q34a

□ Yes, I am already doing so

□ No, school is **not** the place for providing information on vaccine preventable diseases.

□ Prefer not to answer

**Q34a: If yes, what kind of additional information do you need in order to educate students?**

□ Disease specific information (e.g. biology of diseases)

□ History of vaccine preventable diseases (e.g. eradication of small pox)

□ How immunizations work

□ How immunizations protect against disease

□ Safety of immunizations

□ The recommended immunization schedule

□ Rules and regulations surrounding children’s ability to make their own decisions regarding immunization

□ Other

□ Prefer not to answer

**Q35: Do you think information about immunizations should be part of the curriculum?**

□ Yes, but I don’t have enough background 🡪 Go to Q35a

□ Yes, I am already doing so

□ No, school is not the place for information on immunizations

□ Prefer not to answer

**Q35a: If yes, what kind of additional information do you need in order to educate students?**

□ How immunizations work

□ How immunizations protect against disease

□ Safety of immunizations

□ The recommended immunization schedule

□ Rules and regulations surrounding children’s ability to make their own decisions regarding immunization

□ Other

□ Prefer not to answer

*We would like to learn more about your role in the school-based immunization program.*

*For the following statements, please indicate on a scale of 1 – 5, with 1 being strongly disagree and 5 being strongly agree:*

**Q36: My role in the BC school-based immunization program is to distribute and collect consent forms.**

[*5 point scales 1 strongly disagree, 3 neutral and 5 strongly agree]*

**Q37: My role in the BC school-based immunization program is to managing student flow on immunization days**

[*5 point scales 1 strongly disagree, 3 neutral and 5 strongly agree]*

**Q38: My role in the BC school-based immunization program is to inform *students* about the *logistics* of school immunization clinic days (*e.g. when, where, and how the school clinic will run*).**

[*5 point scales 1 strongly disagree, 3 neutral and 5 strongly agree]*

**Q39: My role in the BC school-based immunization program is to inform *students* about the *vaccines* being administered at the school immunization clinic day.**

[*5 point scales 1 strongly disagree, 3 neutral and 5 strongly agree]*

**Q40: My role in the BC school-based immunization program is to inform *parents* about the *logistics* of school immunization clinic days. (*e.g. when, where, and how the school clinic will run*).**

[*5 point scales 1 strongly disagree, 3 neutral and 5 strongly agree]*

**Q41: My role in the BC school-based immunization program is to inform *parents* about the *vaccines* being administered at the school immunization clinic days.**

[*5 point scales 1 strongly disagree, 3 neutral and 5 strongly agree]*

**Q42: I have clear understanding of my role in supporting the school-based immunization program and clinics.**

[*5 point scales 1 strongly disagree, 3 neutral and 5 strongly agree]*

**Q43: Who informed you about what your role is in the school-based immunization program? (Select all that apply)**

- - Public health unit
  - School nurse
  - School District
  - My principal
  - BC Teacher’s Federation (BCTF)
  - Other:
  - I don’t have a role in the school-based immunization program

**Q44: It is appropriate for me as a teacher for me to share my personal opinions about immunizations with my students.**

[*5 point scales 1 strongly disagree, 3 neutral and 5 strongly agree]*

**Q45: It is appropriate for me as a teacher for me to share my personal opinions about immunizations with parents.**

[*5 point scales 1 strongly disagree, 3 neutral and 5 strongly agree]*

**Section – Teaching demographics**

**Q46: What area of study did you complete your undergraduate degree? Select all that apply.**  □ Arts/Social Sciences/Humanities

□ Business/Commerce

□ Engineering

□ Science

□ Other, please specify….

**Q47: In the last five years have you taught (select all that apply):**

□ Grade 6

□ Grade 9

□ I have not taught Grade 6 or 9

□ Prefer not to answer

**Q48: Are you involved in providing sexual education within your currently assigned school?**

□ Yes

□ No

□ Prefer not to answer

**Section - Socio-demographics**

*In the last section of questions, we would like to learn more about you for statistical purposes.*

**Q49: What is your current age in years?**

□**20 - 24**

□**25-29**

□**30-34**

□**35-39**

□**40-44**

□**45-49**

□**50-54**

□**55 - 59**

□**60 - 64**

□**65+**

□ Prefer not to answer

**Q50: What best describes your current gender identify?**

Woman

Man

Trans women

Trans man

Non-binary or gender fluid

Two spirited

I identify as______

Prefer not to answer

**Q51: What sex were you assigned at birth, meaning on your original birth certificate:**

□ Female

□ Male

□ Prefer not to answer

**Q52: Where were you born:** □ In Canada

□ Outside of Canada 🡪 specify country

□ Prefer not to answer

**Q53: How long have you lived in British Columbia?**

□ All my life

□ Less than 2 years

□ Between 2 and 5 years

□ Between 6 and 10 years

□ More than 10 years

□ Not sure

□ Prefer not to answer

**Q54: Do you identify as an Indigenous person from North America?**

- No, I do not identify as an Indigenous person from North America (🡪Go to Q55)
- Yes, First Nations (🡪 Go to Q56)
- Yes, Métis (🡪 Go to Q56)
- Yes, Inuk (Inuit) (🡪 Go to Q56)
- Prefer not to answer (🡪 Go to Q55)

**Q55: Do you consider yourself to be (Check all that apply):**

- White
- South Asian (e.g., East Indian, Pakistani, Sri Lankan, etc.)
- Chinese
- Black
- Filipino
- Latin American
- Arab
- Southeast Asian (e.g., Vietnamese, Cambodian, Malaysian, Laotian, etc.)
- West Asian (e.g., Iranian, Afghan, etc.)
- Korean
- Japanese
- Indigenous person (but do not identify as First Nations, Métis, or Inuk (Inuit))
- I am (please specify): _____
- Prefer not to answer

**Q56: How would you describe your religious or spiritual background, or affiliation, if any?**

- Protestant (for example, United Church of Canada, Anglican, Baptist, Lutheran, Presbyterian, Seven Day Adventist)
- Catholic
- Other Christian
- Christian Orthodox (for example Greek or Russian Orthodox)
- Muslim
- Sikh
- Hindu
- Buddhist
- Jewish (for example, Orthodox, Conservative, Reform, Reconstructionist, or other denomination)
- No religious affiliation
- Other (specify)__________
- Prefer not to answer [skip to next section 🡪]

**Q56a: How much do you agree with the following statement: my religious or spiritual belief system guides my health decisions, including immunization.**

[5-point Likert scale 1 - strongly disagree, 3 - neutral, 5 - Strongly agree]

**Section – COVID-19 vaccine**

*Given the extraordinary times of the COVID-19 pandemic, we would like to ask you a few questions about your opinions and thoughts on a future COVID-19 vaccine.*

*For the following statements, please indicate on a scale of 1 – 5, if you are likely or unlikely:*

**Q57: COVID-19 is a serious disease**

[5 point Likert scale, 1 - Highly disagree, 3 - Neither disagree or agree, 5 - Highly agree]

**Q 58: COVID-19 pandemic has changed the way I feel about immunizations in general**

[5 point Likert scale, 1 - Highly disagree, 3 - Neither disagree or agree, 5 - Highly agree]

**Q59: If a safe and effective COVID-19 vaccine were to become available to the public and recommended for you, how likely are you to receive it?**

[5 point Likert, 1 - very unlikely, 3 - neutral, 5 - very likely]

**Q60: If a safe and effective COVID-19 vaccine were to become available to the public and recommended for children, how likely are you to recommend it to parents/students in your class?**

[5 point Likert, 1 - very unlikely, 3 - neutral, 5 - very likely]

**Q61: We are now at the end of the survey, is there anything else you would like to share with us about your experience with the BC school-immunization program? (open textbox)**

**[SURVEY COMPLETE]**
