# Supplementary material for: Characterization of vaccine confidence among teachers in British Columbia, Canada: A population-based survey
Source: PLoS One. 2023 Jul 12;18(7):e0288107. doi: 10.1371/journal.pone.0288107 (PMC10337953; doi:10.1371/journal.pone.0288107)
Supplement: S1 Table — (As of May 2021). (DOCX) [file pone.0288107.s002.docx]

**S1 Table: BC Immunization Schedule**. As of May 2021.
